# NK cell marker gene-based model shows good predictive ability in prognosis and response to immunotherapies in hepatocellular carcinoma

**DOI:** 10.1038/s41598-023-34602-0

**Published:** 2023-05-05

**Authors:** Juan Li, Yi Li, Fulei Li, Lixia Xu

**Affiliations:** Department of Infectious Diseases, The First Affiliated Hospital of Zhengzhou University, No. 1 Jianshedong Road, Erqi District, Zhengzhou, 450052 China

**Keywords:** Cancer, Oncology

## Abstract

Hepatocellular carcinoma (HCC) is the fourth leading cause of malignancy worldwide, and its progression is influenced by the immune microenvironment. Natural killer (NK) cells are essential in the anti-tumor response and have been linked to immunotherapies for cancers. Therefore, it is important to unify and validate the role of NK cell-related gene signatures in HCC. In this study, we used RNA-seq analysis on HCC samples from public databases. We applied the ConsensusClusterPlus tool to construct the consensus matrix and cluster the samples based on their NK cell-related expression profile data. We employed the least absolute shrinkage and selection operator regression analysis to identify the hub genes. Additionally, we utilized the CIBERSORT and ESTIMATE web-based methods to perform immune-related evaluations. Our results showed that the NK cell-related gene-based classification divided HCC patients into three clusters. The C3 cluster was activated in immune activation signaling pathways and showed better prognosis and good clinical features. In contrast, the C1 cluster was remarkably enriched in cell cycle pathways. The stromal score, immune score, and ESTIMATE score in C3 were much higher than those in C2 and C1. Furthermore, we identified six hub genes: CDC20, HMOX1, S100A9, CFHR3, PCN1, and GZMA. The NK cell-related genes-based risk score subgroups demonstrated that a higher risk score subgroup showed poorer prognosis. In summary, our findings suggest that NK cell-related genes play an essential role in HCC prognosis prediction and have therapeutic potential in promoting NK cell antitumor immunity. The six identified hub genes may serve as useful biomarkers for novel therapeutic targets.

## Introduction

Hepatocellular carcinoma (HCC) has become the most common gastrointestinal tumor globally with an increasing incidence and high mortality rate^1^. The initiation and progression of HCC is an extremely complicated and gradual process involving multiple factors, stages and genetic mutations^2^. The molecular mechanism of tumorigenesis and development is intricate and is yet to be comprehensively understood. A growing number of studies have focused on the regulation of immune cell-mediated functions in the tumor microenvironment (TME) of HCC^3^. The TME in HCC has prominent roles in tumor initiation and progression, and the relevant immune features have been defined^4,5^. Several literatures support that genomic signatures have strong correlations with response to immune checkpoint inhibitors, which might provide evidence of available biomarkers to guide clinical decision-making^3^.

Natural killer (NK) cells have been reported as important members of the innate immune system, which are involved in direct tumor cell killing in response to immunotherapies^6^. They are an attractive therapeutic target in liver disease due to their role in immunosurveillance and their ability to recognize and eliminate malignant cells^4^. NK cells are also particularly enriched in the liver and have been recognized to contribute to the pathogenesis of liver cancer^7^. NK cells are involved in anti-tumor immunity by directly killing tumor cells and enhancing the adaptive T cell activity to compromise the immune killing function of tumor cells^8^.

NK cell-based immunotherapies cannot completely achieve human leukocyte antigen matching and have fewer side effects^9^. It was originally thought that NK cells kill cancer cells mainly through apoptosis^10^. Meanwhile, studies on NK cell homeostasis and function suggested a potential strategy for invigorating NK cell-based immunotherapy^11^. Researches had figured out the notion that the combination of BH3 mimetics and NK cells might aggravate the tumor cell killing ability^12^. In a recent study, the use of NK cells for the generation of CAR effector cells has been shown to be safe and effective, offering potential advantages^13^. State-of-the-art research focuses on the underlying mechanisms of NK cells in immunity and the molecular characteristics of NK cells in cancers^14^, whereas the internal molecular mechanisms of NK cells in HCC are relatively poorly known.

In the current study, we investigated the genomic expression of NK cell-related genes. Based on their expression level, samples were classified into clusters, which had significant differences in clinical and pathologic features and immune microenvironment signatures. In addition to the expression of immune checkpoint genes, immune cell infiltrations were also explored. Our study might provide clues for targeted therapeutics in HCC.

## Results

### Molecular classification based on NK cell-related genes

**Figure 1:**
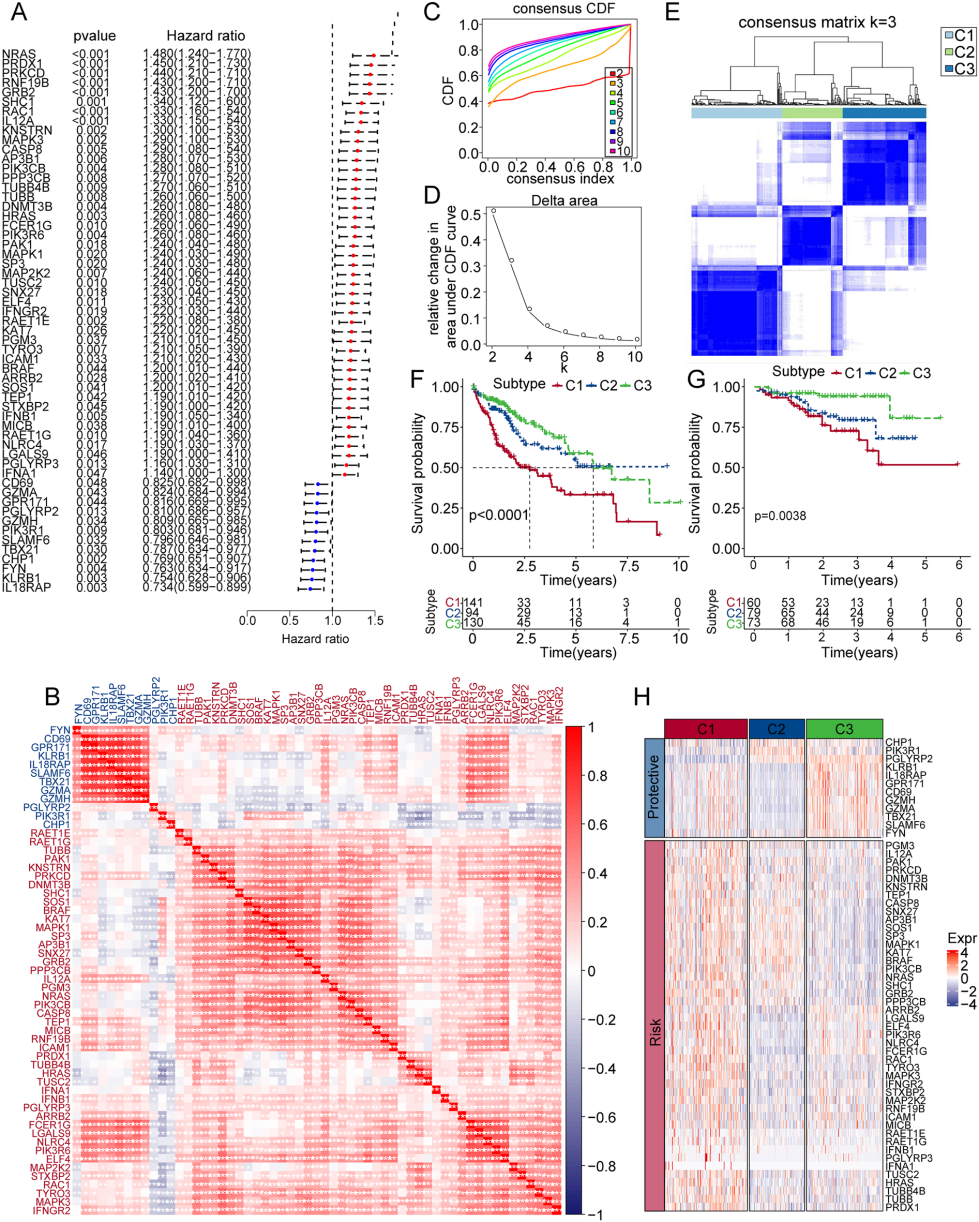
Identification and classification of NK cell-related genes. (**A**) Forest map of natural killer cell-associated genes that significantly correlated with HCC prognosis. (**B**) Univariate Cox correlations of significant natural killer cell gene expression in TCGA-LIHC. (**C**) CDF delta area curves of TCGA-LIHC cohort samples. (**D**) Relative changes in the area under CDF curve. (**E**) Heatmap of consensus k = 3. (**F**) Overall survival time curve between 3 clusters. (**G**) Prognostic differences among the three molecular subtypes in the ICGC cohort. (**H**) Heatmap suggesting the expression of natural killer cell-related genes with significant prognosis in 3 clusters of TCGA-LIHC.

In order to explore the NK cell-related gene signatures, we adopted the expression level of these genes and the R package ‘coxph’ with univariate Cox regression analysis, and found 57 significant HCC prognosis-related genes (Fig. 1A). 12 NK cell related genes were negatively correlated with HCC prognosis, and 45 NK cell related genes had positive correlation. Next, we analyzed the correlations between these 57 genes, and found that they were closely related (Fig. 1B). We then performed consistency cluster analysis on the 57 NK cell-related genes. The consensus cumulative distribution function (CDF) curve (Fig. 1C) and the change in area under CDF delta area curve (Fig. 1D) showed that, for consensus matrix k = 3, NK cell-related gene-based classification had relatively stable clustering results. Three clusters such as cluster 1 (C1), cluster 2 (C2) and cluster 3 (C3) were clearly identified (Fig. 1E). To better explore the prognosis features of these clusters, we adopted Kaplan–Meier analysis. We figured out that the overall survival time of the C3 cluster was the best, and the C1 cluster had poor prognosis compared with C1 and C3 (Fig. 1F). We then performed the same methods on the ICIC cohort, and similarly identified three clusters. The C1 cluster had better prognosis compared with C3 (Fig. 1G). In addition, we compared the expression level of 57 NK cell-related genes between the 3 clusters. We found out that these prognosis risk genes were generally highly expressed in the C1 cluster, whereas the prognosis-related protective genes were highly expressed in the C3 cluster (Fig. 1H).

### Genomic alterations and mutational signatures between the 3 clusters

To better understand the genomic profile signatures between the established 3 clusters, we performed the comparison of molecular features, and found that the C1 cluster had higher aneuploidy score, homologous recombination deficiency, intratumor heterogeneity, loss of heterozygosity, and ploidy (Fig. S1A–E). The TMB exhibited no significant differences between the 3 clusters (Fig. S1F). Among the 3 clusters, C2 had higher purity level compared with C1, and C3 had lower purity level compared with C2 and C1 (Fig. S1G). Next, we classified HCC patients into 5 subgroups (1–5) based on their immune signatures. We further compared the proportion of immune subgroups in each cluster and validated that the proportion of immune signatures-based subgroups in C1 was significantly different compared with C3 (Fig. S1H). We also compared the correlation between gene mutations and molecular subtypes and found out significant difference between subtypes. The TP53, LRP1B and other genes had extensive somatic mutations in HCC (Fig. S1I).

### Differences in signaling pathways among the 3 clusters

To better explain the enriched signaling pathways in NK cell-related gene-based clusters, we applied the Hallmark database and its candidate genes with GSEA. The results indicated that 14 signaling pathways were markedly enriched in the C1 cluster compared with the C3 cluster in the TCGA-LIHC cohort. Meanwhile, in the ICGC cohort, there were 30 enriched signaling pathways, such as MYC TARGES, E2F TARGETS, G2M CHECKPOINT, MYC TARGETS-V2, DNA REPAIR, etc. (Fig. S2A). Next, we compared the 3 clusters for differences in signaling pathways. The results demonstrated that C1 was remarkably enriched in cell cycle pathways, and C3 was activated in immune activation signaling pathways (Fig. S2B). Therefore, NK cell-related genes might play very important roles in the cell cycle and tumor immune microenvironment activation.

### Immune signatures between the 3 clusters

**Figure 2:**
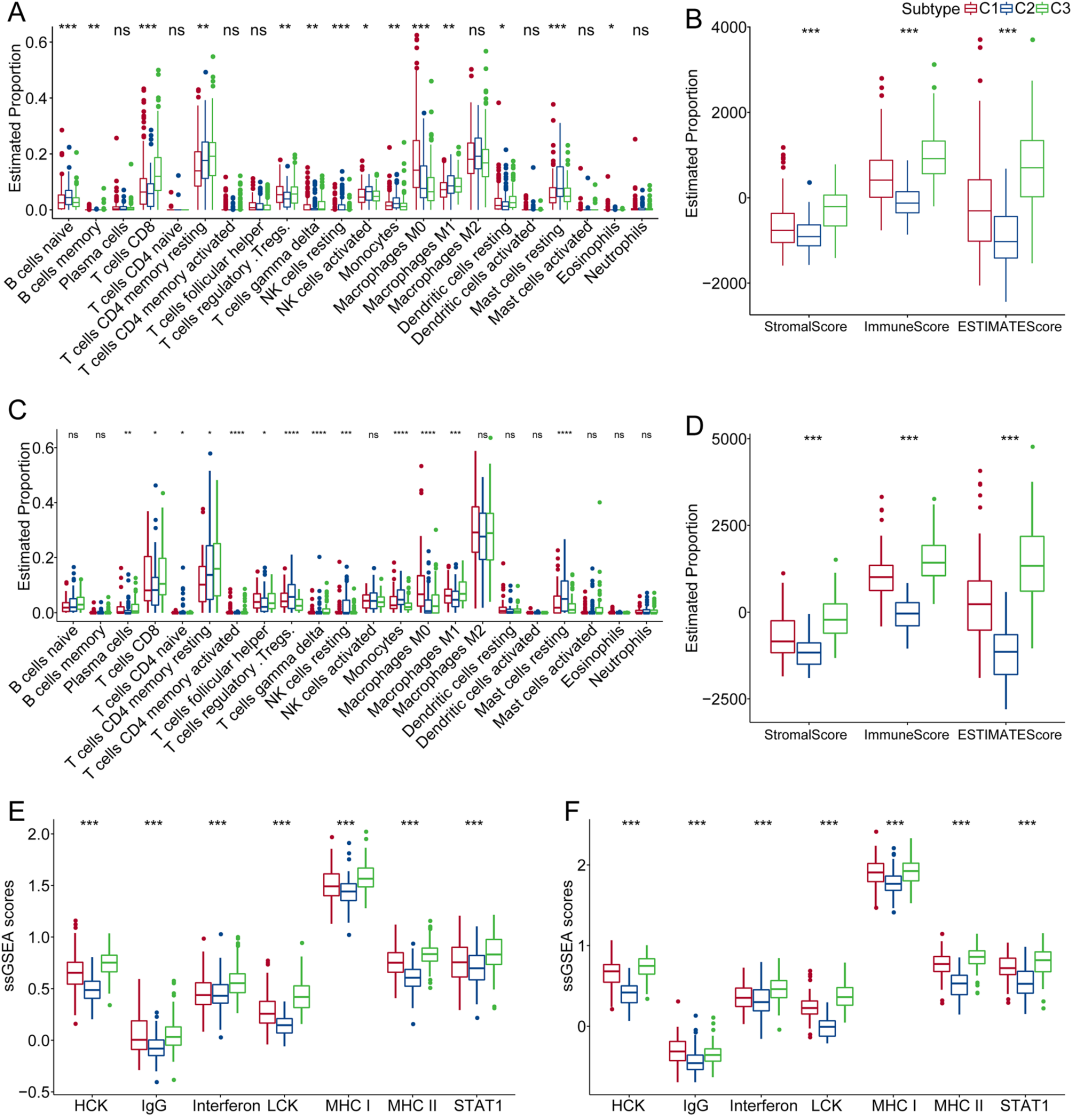
Infiltration level of 22 types of immune cells in two hepatocellular carcinoma cohorts. (**A**) Differences in 22 immune cell scores among different molecular subtypes in the TCGA-LIHC cohort. (**B**) Estimation of the differences in immune infiltration between different molecular subtypes in the TCGA-LIHC cohort. (**C**) Differences in 22 immune cell scores among different molecular subtypes in the ICGC cohort. (**D**) ESTIMATE in the CGC cohort illustrated the difference in immune infiltration between different molecular subtypes. (**E**) Differences in the scores of seven inflammatory gene clusters among different molecular subtypes in the TCGA-LIHC cohort. (**F**) Differences in the scores of seven inflammatory gene clusters among different molecular subtypes in the ICGC cohort.

To better clarify the differences in tumor immune microenvironment between the 3 established clusters, we investigated the gene expression and infiltration level of immune cells by CIBERSORT and ESTIMATE. The results suggested that the infiltration level of most immune cells was significantly different between the 3 clusters except for plasma cells, CD4 T cells, activated CD4 memory T cells, helper follicular T cells, M2 macrophages, resting dendritic cells, activated mast cells, and neutrophils (Fig. 2A). The ESTIMATE results showed that the stromal score, immune score, and ESTIMATE score in the C3 cluster were much higher than those in the C2 and C1 cluster (Fig. 2B). In addition, we compared the immune infiltration of the validation dataset with that of the ICGC cohort, and the latter had similar results as the TCGA cohort (Fig. 2C,D). Furthermore, we analyzed the inflammatory activity of the three clusters. The enrichment scores of the seven Meta-genes were significantly different among the three clusters. The C1 cluster had higher inflammatory activity, followed by C1, as shown in Fig. 2E, and this phenomenon was also observed in the ICGC cohort (Fig. 2F).

### Evaluation of the expression of immune checkpoint genes and immunotherapy response between the 3 clusters

**Figure 3:**
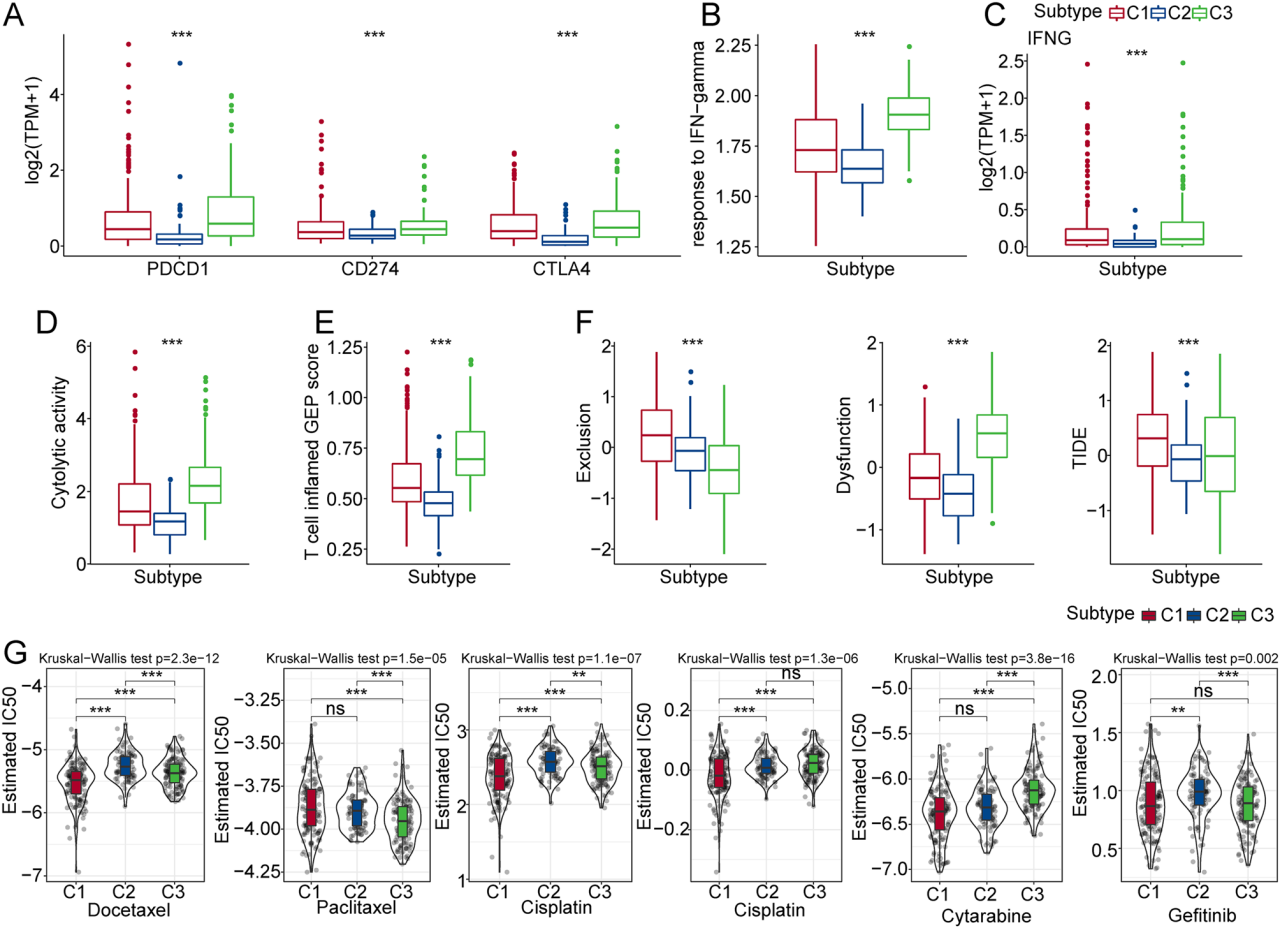
Evaluation of immune-related indicators between the 3 clusters. (**A**) Differences in immune checkpoint gene expression among the 3 clusters. (**B**) Differences in “T cell inflamed GEP score” among the 3 clusters. (**C**) Differences in response to IFN − gamma among the 3 clusters. (**D**) Differences in the expression of INFG gene in the 3 clusters. (**E**) Differences in “Cytolytic activity” between the 3 clusters. (**F**) Differences in the TIDE scores of the 3 clusters. (**G**) The box plots of the estimated IC50 for docetaxel, paclitaxel, cisplatin, cytarabine, bortezomib, and gefitinib in the 3 clusters from the TCGA-LIHC cohort.

Immune checkpoint blockade-mediated immunotherapy had shown promising effects against tumors, and had proved as effective in a significant proportion of refractory patients undergoing standard therapy. In this analysis, we evaluated some representative targets, and found that PDCD1, CD274 and CTLA4 were highly expressed in the C3 cluster (Fig. 3A).

The results supported that IFN-γ response was notably higher in C1 compared with C2 and C3 (Fig. 3B). We then compared the expression differences in INFG genes between the three subtypes and found out that INFG was significantly overexpressed in C3 (Fig. 3C). The cytolytic activity score in the C3 cluster was much higher compared with the other 2 clusters (Fig. 3D). The T cell inflamed GEP score showed a similar trend between the 3 clusters (Fig. 3E). We further adopted TIDE software and evaluated the potential effect of immunotherapies. The results demonstrated that C1 had the highest score in exclusion and TIDE compared with C2 and C3. The dysfunction score of C1 was much lower compared with C3 (Fig. 3F). A higher TIDE score represents higher immune escape and lower effectiveness in immunotherapies. Next, we evaluated the response of different clusters to chemotherapies. Traditional chemotherapy drugs included docetaxel, paclitaxel, cisplatin, cytarabine, bortezomib, and gefitinib in the TCGA-LIHC cohort. The results indicated that C1 was found to be more sensitive to docetaxel, cisplatin, cytarabine, bortezomib, and gefitinib (Fig. 3G).

### Identification of hub genes among the NK cell-related genes and risk model construction

**Figure 4:**
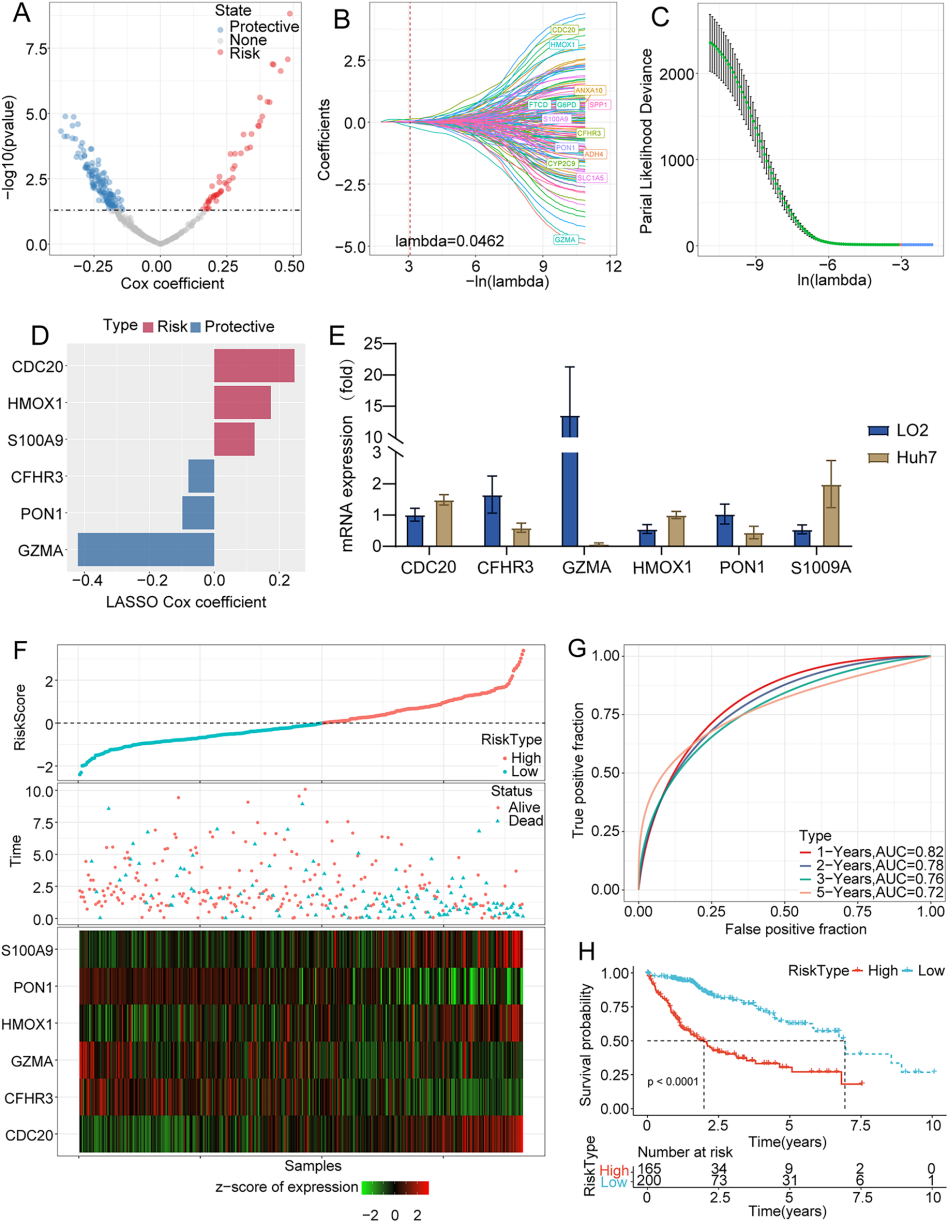
Identification of hub genes and risk model construction. (**A**) A total of 183 promising candidates were identified among the DEGs. (**B**) Trajectories of each independent variable with lambda. (**C**) The confidence interval under lambda. (**D**) The distribution of LASSO coefficients of the Natural Killer cell-related gene signature. (**E**) RNA expression level between the Huh7 cell line and the LO2 cell lines. (**F**) Risk score, survival time and survival status, and the expression of necroptosis-related genes in the TCGA-LIHC dataset. (**G**) ROC curve and the AUC of risk score classification in the TCGA-LIHC dataset. (**H**) Kaplan–Meier survival curve distribution of risk score in the TCGA-LIHC dataset.

In the previous section, we have identified 3 clusters. The ‘limma’ R package was applied to screen out the DEGs in C1 versus others, C2 vs others and C3 vs others (FDR < 0.05 and |log2FC|> 1). Overall, we recognized 345 DEGs between different clusters. Next, we used univariate COX regression analysis on the DEGs and identified 183 genes with significant prognostic effects (*p* < 0.05), including 49 “risk” genes and 134 “protective” genes (Fig. 4A). These 183 genes with significant prognosis were further compressed using the R package ‘glmnet’ for LASSO regression analysis, to minimize the number of hub genes for risk score model construction. From the change trajectory of prognostic precursor genes of each independent variable, it could be seen that, with the gradual increase in lambda, the number of independent variable coefficients tending to 0 also gradually increases. Then, we applied tenfold cross-validation to construct the model and analyzed the confidence intervals under each lambda. The results suggested that the model reached the optimum for lambda = 0.0462. Therefore, we selected 13 genes for this scenario as the target genes for the next step (Fig. 4B). Based on these 13 genes from the LASSO analysis results, we further used stepwise multivariate regression analysis. In this step, the AIC Akaike information criterion was used, and one variable was successively deleted to reduce AIC, to obtain the best fit degree (Fig. 4C). Ultimately, we identified six genes as key genes associated with natural killer cells that influence prognosis (Fig. 4D). We also adopted the qPCR analysis, and figured out that CDC20, HOMX and S1009A were significantly highly expressed in HCC cell lines compared with LO2 cell lines. Meanwhile, CFHR3, GZMA, and PON1 were lower expressed in Huh7 cell lines compared with LO2 cell lines (Fig. 4E).

Further better understanding the prognostic model based on 6 NK cell-related genes, we calculated the prognostic risk score of each sample and normalized it. The distribution of risk score of each sample in the TCGA-LIHC cohort was shown in Fig. 4F. According to the risk score distribution, survival status and heatmap of gene expression, the high and low risk score subgroups had significant differences. The 1 year, 2 years, 3 years, and 5 years AUC of the prognostic model based on these 6 genes exhibited a high AUC area, suggesting that this model had stable and good overall performance (Fig. 4G). The overall survival time curve showed that low the risk score subgroup had longer survival time (Fig. 4H).

### The risk score combined with clinicopathological features further improves the prognostic model and survival prediction

**Figure 5:**
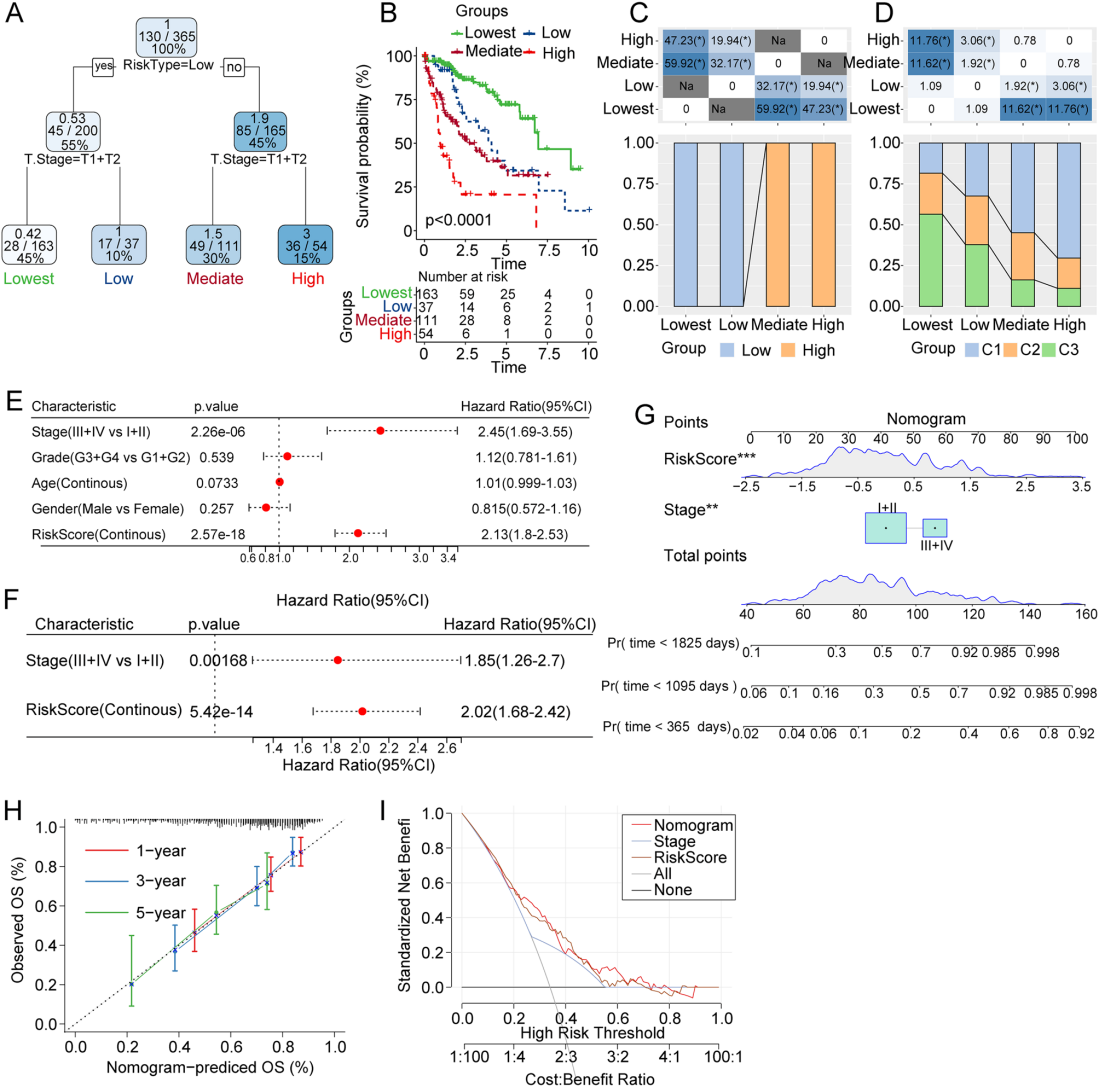
Model construction and validation based on risk score and clinical signatures. (**A**) Patients with full-scale annotations, including risk score, age, gender, and TNM Stage, were used to build a survival decision tree to optimize risk stratification. (**B**) Significant differences of overall survival were observed among the four risk subgroups. (**C**, **D**) Comparison and analysis between different groups. (**E**, **F**) Univariate and multivariate Cox analysis of risk score and clinicopathological characteristics. (**G**) The nomogram model based on risk score and stage. (**H**) Calibration curves of nomogram for years 1, 3 and 5 based on this risk score and clinical feature-based model. (**I**) Decision curve of nomogram with all factors.

To comprehensively evaluate the model, we constructed a decision tree based on the age, gender, TNM stage pathological information, and risk score of patients in the TCGA-LIHC cohort. The results indicated that risk score and T stage were the only key factors in the decision tree, and four different risk subgroups were identified as “lowest”, “low”, “medium”, and “high” subgroups (Fig. 5A). The Kaplan–Meier curve highlighted that the lowest risk subgroup showed the highest survival probability (Fig. 5B). The proportion of high and low risk score subgroups among these four subgroups indicated that low risk score samples were mainly located in high and medium subgroups and had significant differences compared with the high-risk score subgroup mainly located in the low and lowest groups (Fig. 5C). The proportions of C1, C2 and C3 clusters were significantly different for the four subtypes (Fig. 5D). We adopted the univariate and multivariate cox regression analysis of risk score and clinicopathological characteristics, and the results showed that the clinical stage and risk score were the most significant prognostic factors (Fig. 5E–G). Furthermore, the calibration curve was used to evaluate the prediction accuracy of the model. The calibration curves from the points of 1, 3, and 5 years were close to the standard curve, indicating that the nomogram had good prediction performance (Fig. 5H). The reliability of the model was also evaluated using the decision curve, and it was observed that both the risk score and the nomogram had significantly higher benefits than the extreme curves. To quantify the risk assessment and survival probability of patients with liver cancer, we developed a nomogram combining the risk score with other clinicopathological features to demonstrate that the risk score had the greatest impact on survival prediction (Fig. 5I).

### Clinical features between high and low risk score subgroups

**Figure 6:**
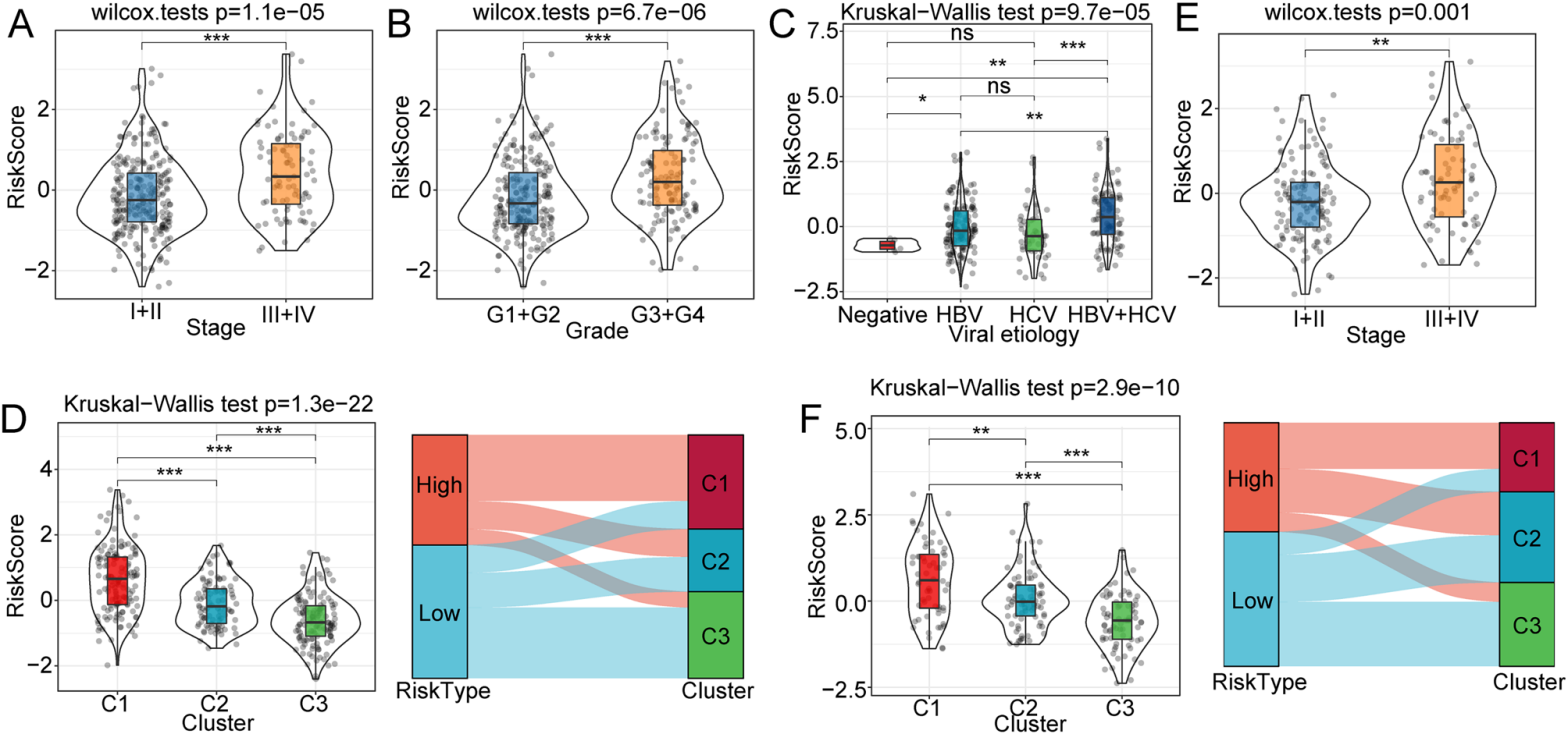
Differences in clinical features between high and low risk score subgroups. (**A**) Risk score differences among different tumor stages in the TCGA-LIHC cohort. (**B**) Risk score differences in tumor grades in the TCGA-LIHC cohort. (**C**) Risk score differences between different viral groups in TCGA-LIHC cohort. (**D**) Risk score differences among 3 clusters in the ICGC cohort. (**E**, **F**) Risk score differences between different tumor stages and grades in the ICGC cohort.

In order to better explore the clinical characteristics between high and low risk score subgroups, our analysis revealed that high risk score subgroup samples had advanced tumor stages and tumor grades (Fig. 6A,B). The advanced viral etiology had higher risk score level (Fig. 6C). The NK cell-related gene-based 3 clusters had remarkably different risk score levels: the C1 cluster had a significantly higher risk score compared with C2 and C3 (Fig. 6D). We further investigated the distribution of risk score subtypes within the 3 clusters, and figured out that most samples with low risk score were also located in C2 and C3 (Fig. 6D). Similar results were shown for the ICGC cohort (Fig. 6E,F). Taken together, our findings revealed the clinical features between high and low risk score subgroups.

### Immune cell infiltration level, enriched signaling pathways, and response to immunotherapies and chemiotherapies between high and low risk score subgroups

**Figure 7:**
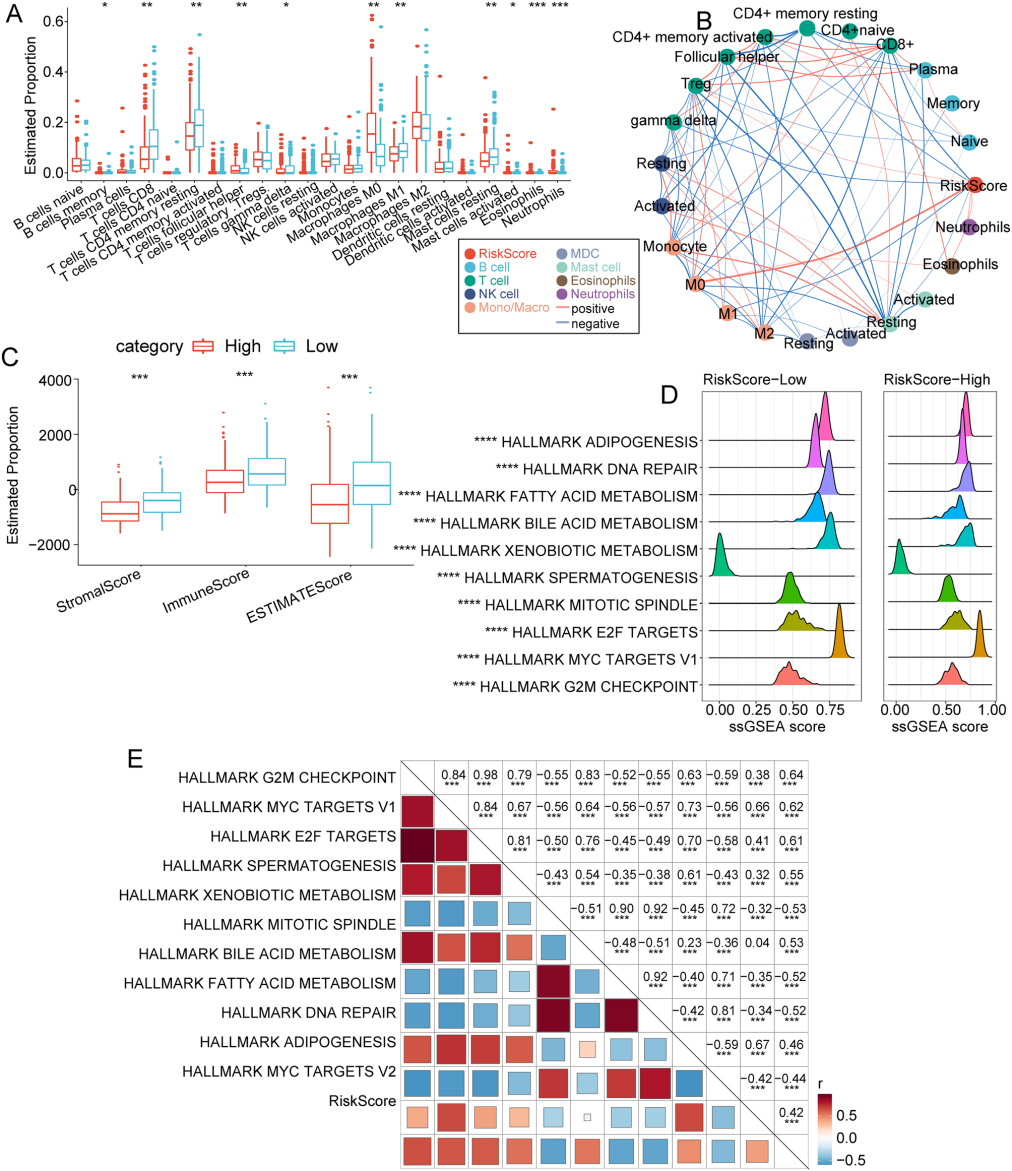
Immune cell infiltration and immune-related indicators between high and low risk score subgroups. (**A**) Proportion of immune cell components in the TCGA cohort. (**B**) Correlation analysis between 22 immune cell components and risk score in the TCGA cohort. (**C**) Proportion of immune cell components calculated by the ESTIMATE software in the TCGA cohort. (**D**) Top 10 pathways with the most significant differences between high and low risk score. (**E**) Correlation analysis between KEGG pathways with correlations greater than 0.4.

To clarify the differences in immune microenvironment between the groups, we compared the infiltration level of 22 types of immune cells and figured out that 11 of them were significantly different, as shown in Fig. 7A. The internal correlations between these 22 immune cells were intricate (Fig. 7B). We also adopted the ESTIMATE software to assess the immune cell infiltration level. The results showed that the low risk score subgroup had higher immune cell infiltration level (Fig. 7C). Then, we adopted the ssGSEA and GSEA to investigate the enriched signaling pathways between high and low risk score subgroups. The high risk score subgroup was mainly enriched in cell cycle-related signaling pathways (Fig. 7D). Furthermore, the correlation between the enrichment scores of these functions and risk score was calculated, and the functions with a correlation greater than 0.4 were selected. These evidences supported that the risk score was positively correlated with cell cycle-related pathways (Fig. 7E). Collectively, our results revealed the differences in immune cell infiltration level, enriched signaling pathways and response to immunotherapies and chemotherapies between groups.

## Discussion

Cancers are a group of malignancies that remain the leading cause of death worldwide^15^. With the difficulties of early diagnosis and insufficient efficacy of advanced-stage therapies, the main obstacles for cancer management are exploring novel diagnostic biomarkers for early diagnosis and improving therapy effectiveness and prognosis prediction^16^. The liver is the largest immune organ that has typical immune tolerance characteristics^17^. HCC has become the leading cause of cancer-related deaths in the world^18^. It has a complex ecosystem featured by high heterogeneity^19^. In recent years, the roles of the immune system in hepatic physiology and the pathological changes during tumor HCC initiation and progression have been increasingly recognized. HCC are distinct from other cancers in that it typically features a status of long-term low-grade inflammation^20^. Investigating the internal mechanisms that underlie inflammation-driven tissue remodeling of the hepatic immune environment might help to provide novel approaches into much needed treatments for this devastating disease. Immune cells in the liver play crucial roles in the initiation and progression of HCC^4^. NK cells, a group of adaptive immune cells, are abundantly enriched in the liver and thought to contribute to the pathogenesis of liver cancer^21^. Hence, they are potential therapeutic targets, albeit underexplored given their important role in immunosurveillance and their capability to recognize and eliminate tumor cells^4^.

Traditional approaches include chemotherapies, such as sorafenib or lenvatinib that are still listed as first-line drugs. In recent years, the immune-checkpoint inhibitors (ICIs) have revolutionized cancer therapy in unsolid tumors. They have shown promising effects against tumors and proven as effective in a significant proportion of refractory patients undergoing standard therapy^22^. Systematic evaluation of the immune microenvironment provides alternative immunotherapeutic tools that could help overcome their limitations. NK cells have great anti-tumor ability in HCC, initiating T cell response by chemical signals and also enhancing the density of immune cells. Moreover, accumulating evidence has demonstrated that NK cell activities are also involved in tumor progression^4^. The melanoma cell was often reported as insufficient to inhibit NK cell-mediated cytotoxicity. Studies also figured out that metastatic melanoma biopsies of pembrolizumab responders had higher NK cell infiltration level compared with non-responders, which was also remarkably correlated with dendritic cell infiltration^23^. The infiltration of NK cells is also closely associated with tumor prognosis: higher NK cell infiltration levels predict better prognosis in squamous cell lung carcinoma, gastric cancer, and colorectal carcinoma^24^. Further exploring the immune environment in HCC from multiple perspectives might pave ways to derive novel therapeutic approaches, identify novel biomarkers, assess pre-treatment effects and predict the post-treatment prognosis risk.

In this analysis, we compared the NK cell-related gene expression levels of liver cancer patients from public databases. We identified 3 clusters, among which the C3 cluster had better prognosis and C1 had the worst prognosis. For immune subtype classification, we figured out that there was a significant difference in immune-related evaluation, clinical pathological signatures, and genomic mutation landscapes. These results demonstrated that distinguishing between NK cell-related gene-based clusters provides a novel classification avenue for HCC. In addition, we identified 6 hub genes, namely CDC20, HMOX1, S100A9, CFHR3, PCN1, and GZMA, for risk score evaluation and prognosis prediction. We constructed a risk score model and figured out that a higher risk score suggests poor prognosis compared with a lower risk score. In addition to clinical features, immune infiltration level and enriched signaling pathways also showed significant differences between high and low risk score subgroups, helping to predict the prognosis and the effects of chemotherapy and immunotherapy.

In conclusion, NK cell-related gene signature-based classification and NK gene-related risk score model can facilitate prognosis prediction and the selection of individualized immunotherapeutics for HCC patients.

## Materials and methods

### Database sources

In this analysis, we adopted The Cancer Genome Atlas (TCGA) public database and the Genomic Data Commons (GDC) API to download the RNAseq data of TCGA-LIHC (https://portal.gdc.cancer.gov/). After screening, a total of 365 primary tumor samples were included. We downloaded the information of International Cancer Genome Consortium (ICGC) LIRI-JP cohort via the Hepatocellular Carcinoma (HCCDB, http://lifeome.net/database/hccdb/home.html) database. From this source, 212 liver cancer samples were included. We also adopted the Gene Expression Omnibus (GEO, https://www.ncbi.nlm.nih.gov/geo/query/acc.cgiacc=GSE14520), which contained transcriptome profiles of HCC samples and corresponding adjacent normal liver tissues. And 221 liver cancer samples were included in this analysis. We recognized the TCGA-LIHC cohort as the training database, while the ICGC-LIRI-JP and GSE14520 cohorts served as validation datasets.

### Data processing

The gene expression profile was measured experimentally using the Illumina HiSeq2000 RNA Sequencing platform and transformed into log2 scale. We adopted patients histologically diagnosed with HCC, with transcriptional profiles, and survival clinical information were included in this analysis. The clinical parameters included gender, age, tumor T N M stages, clinical status, status, and follow-up period. Samples without survival information were excluded.

### Origin of natural killer (NK) cell-related genes

In this analysis, we adopted NK cell genes from the ImmPort Portal (https://www.immport.org/home), which yielded 134 NK cell-related genes for analysis. The 18 NK cell-related pathways were downloaded from the molecular signatures database. In addition, we selected 79 NK cell genes from the LM22 database (LM22 containing 22 functionally defined human immune subsets), which is a validated leukocyte gene signature matrix.

### Data preprocessing

The following steps were performed to preprocess the RNA-seq of TCGA. Samples with insufficient clinical information, or those missing survival time or clinical tumor status were removed. The Ensemble was converted into Gene Symbol. In addition to the GEO dataset, we downloaded the annotation information of the corresponding genomic platform, mapped probes to genes according to the annotation information, and removed probes that matched one probe to multiple genes. When multiple probes matched with a gene, the mean value was taken as the gene expression value.

### Molecular subtyping of NK cell-related genes

In order to better classify the NK cell-related genes into different clusters, we adopted the ConsensusClusterPlus tool to construct the consensus matrix and cluster the NK cell-related expression profile data of samples. We adopted the “KM” algorithm and “1-Pearson correlation”, and applied the metric distance and 500 bootstraps, with each bootstrap process including 80% of patients in the training set. We identified the cluster number as 2–10. To better determine the optimal classification, we calculated the consistency matrix and the consistency cumulative distribution function. We can get better clustering quality when K = 3. Finally, three specified molecular subtypes of the HCC cohorts were obtained.

### Risk score model construction

In order to construct the risk score model, we recognized the differentially expressed NK cell-related genes among the clusters with a false discovery rate (FDR) of 0.05 and |log2FC|> 1. Genes with P less than 0.05 were regarded as significant prognosis-related differentially expressed genes. To reduce the number of genes and construct the prognostic risk model, we performed least absolute shrinkage and selection operator (LASSO) regression analysis, and obtained NK phenotype-related significant prognostic genes. The risk score of each sample was calculated by riskscore = ∑βi × Exp, where “i” represents the gene expression level of NK cell phenotype-related genes, and β represents the Cox regression coefficient of corresponding genes. Based on the threshold of “0”, patients were divided into high and low risk subgroups. We applied the Kaplan–Meier method to draw survival curves, and performed the log-rank test to determine the significance of the differences between clusters.

### Evaluation of immunotherapy response

Tumor Immune Dysfunction and Exclusion (TIDE) is a convenient and efficient method to evaluate the response to immunotherapy based on the gene expression profiles^25^. In this analysis, TIDE was carried out to verify the impact of the efficacy of risk score prediction model on clinical responsiveness to immune checkpoint inhibitors (ICIs).

### Single-sample gene set enrichment analysis (GSEA) and KEGG enrichment

All candidate differentially expressed genes in the Hallmark database were adopted in this analysis. The GSEA analysis was also conducted to explain differences in enriched signaling pathways, especially in immune infiltration and macrophage-associated biological pathways. In addition, the correlation between the KEGG (https://www.kegg.jp/kegg/kegg1.html)^26^ enrichment scores of these functions and risk score was calculated.

### Evaluation of tumor immune microenvironment-related immune cell infiltration

The tumor microenvironment (TME) is key to the pathogenesis of solid tumors. CIBERSORT^27^ (https://cibersort.stanford.edu) was utilized to reveal the immune cell infiltration patterns, determine the proportions of circulating immune cells and analyze the correlation between these immune cells. ESTIMATE provides basic information with tumor purity level, stromal cell infiltration level, and immune cell infiltration level in tumor tissues based on the genomic profiles.

### Evaluation of risk score and chemotherapy response

We employed the ‘pRRophetic’ R package to calculate and evaluate the IC50 and sensitivity of immunosuppressants. This tool has good prediction abilities on clinical chemotherapy response based on gene expression levels. The prediction by ‘pRRophetic’ included drug sensitivity for methotrexate, parthenolide and rapamycin.

### Cell culture, RNA extraction and real time PCR analysis

In this study, we adopted the LO2 cell line and Huh7 cell line and cultured them in 37 °C incubator with Dulbecco modified eagle medium + 10% fetal bovines serum and 1% penicillin streptomycin. The total RNA was extracted by Fastpure cell/tissue total RNA isolation kit V2 (RC112-01, Vazyme, China) according to the instruction. We performed the ChamQ Universal SYBR qPCR Master Mix kit (Vazyme, China) on the reverse transcription the by biosystems (Quant Studio TMDx) machine. In addition, we conducted the real time PCR analysis by fluorescence qPCR instrument (ABI, Quant Studio TMDx, Thermo Fisher Scientific, Inc.). Finally, we calculated the RNA expression level by fold change = 2^−ΔΔCT^. All target genes primers were listed in Table S1.

### Statistical analysis

All statistical analyses were conducted using the R software version 4.0.5. We also used the Student’s t-test to compare the differences between two subgroups, and performed the Kaplan–Meier method to analyze the differences in survival curves using the log-rank test.

### Disclosure

TCGA, HCCB and GEO belong to public databases. The patients involved in the database have obtained ethical approval. Users can download relevant data for free for research and publish relevant articles. Our study is based on open-source data, so there are no ethical issues and other conflicts of interest.

## Supplementary Information

Supplementary Figures.

Supplementary Table S1.

## Data Availability

The datasets used and/or analysed during the current study are available from the public database (TCGA-LIHC, ICGC-LIRI-JP, and GSE14520). And the original contributions presented in the study are included in the article/supplementary material, further inquiries can be directed to the corresponding authors.

## References

[1] Johnson P, Zhou Q, Dao DY, Lo YMD (2022). Circulating biomarkers in the diagnosis and management of hepatocellular carcinoma. Nat. Rev. Gastroenterol. Hepatol..

[2] Calderaro J, Seraphin TP, Luedde T, Simon TG (2022). Artificial intelligence for the prevention and clinical management of hepatocellular carcinoma. J. Hepatol..

[3] Llovet JM (2022). Immunotherapies for hepatocellular carcinoma. Nat. Rev. Clin. Oncol..

[4] Curio S, Belz GT (2022). The unique role of innate lymphoid cells in cancer and the hepatic microenvironment. Cell Mol. Immunol..

[5] Lu Y (2022). Resident immune cells of the liver in the tumor microenvironment. Front. Oncol..

[6] Watkins-Schulz R (2022). Microparticle delivery of a STING agonist enables indirect activation of NK Cells by antigen-presenting cells. Mol. Pharm..

[7] Moretta L (2007). NK cell-mediated immune response against cancer. Surg. Oncol..

[8] Badrinath S (2022). A vaccine targeting resistant tumours by dual T cell plus NK cell attack. Nature.

[9] Delfanti G (2022). TCR-engineered iNKT cells induce robust antitumor response by dual targeting cancer and suppressive myeloid cells. Sci. Immunol..

[10] Yang H, Jia H, Zhao Q, Luo KQ (2022). Visualization of natural killer cell-mediated killing of cancer cells at single-cell resolution in live zebrafish. Biosens. Bioelectron..

[11] Guo X (2022). NAD(+) salvage governs mitochondrial metabolism, invigorating natural killer cell antitumor immunity. Hepatology.

[12] Narni-Mancinelli E, Vivier E (2022). Advancing natural killer therapies against cancer. Cell.

[13] Liu E (2020). Use of CAR-transduced natural killer cells in CD19-positive lymphoid tumors. N. Engl. J. Med..

[14] Ascierto ML (2015). Inherent transcriptional signatures of NK cells are associated with response to IFNα + rivabirin therapy in patients with Hepatitis C Virus. J. Transl. Med..

[15] Eaton JE, Talwalkar JA, Lazaridis KN, Gores GJ, Lindor KD (2013). Pathogenesis of primary sclerosing cholangitis and advances in diagnosis and management. Gastroenterology.

[16] Wang Y (2022). Immune checkpoint modulators in cancer immunotherapy: Recent advances and emerging concepts. J. Hematol. Oncol..

[17] Montella L (2021). The role of immunotherapy in a tolerogenic environment: Current and future perspectives for hepatocellular carcinoma. Cells.

[18] Calderaro J (2022). Nestin as a diagnostic and prognostic marker for combined hepatocellular-cholangiocarcinoma. J. Hepatol..

[19] Liu Y, Zhang L, Ju X, Wang S, Qie J (2022). Single-cell transcriptomic analysis reveals macrophage-tumor crosstalk in hepatocellular carcinoma. Front. Immunol..

[20] Zaki MYW (2022). Innate and adaptive immunopathogeneses in viral hepatitis; Crucial determinants of hepatocellular carcinoma. Cancers.

[21] Qian Y, Shang Z, Gao Y, Wu H, Kong X (2022). Liver regeneration in chronic liver injuries: Basic and clinical applications focusing on macrophages and natural killer cells. Cell Mol. Gastroenterol. Hepatol..

[22] Salem R, Greten TF (2022). Interventional radiology meets immuno-oncology for hepatocellular carcinoma. J. Hepatol..

[23] Barry KC (2018). A natural killer-dendritic cell axis defines checkpoint therapy-responsive tumor microenvironments. Nat. Med..

[24] Krasnova Y, Putz EM, Smyth MJ, Souza-Fonseca-Guimaraes F (2017). Bench to bedside: NK cells and control of metastasis. Clin. Immunol..

[25] Jiang P (2018). Signatures of T cell dysfunction and exclusion predict cancer immunotherapy response. Nat. Med..

[26] Kanehisa M, Furumichi M, Sato Y, Kawashima M, Ishiguro-Watanabe M (2023). KEGG for taxonomy-based analysis of pathways and genomes. Nucleic Acids Res..

[27] Newman AM (2015). Robust enumeration of cell subsets from tissue expression profiles. Nat. Methods.

