# Supplementary material for: NK cell marker gene-based model shows good predictive ability in prognosis and response to immunotherapies in hepatocellular carcinoma
Source: Sci Rep. 2023 May 5;13:7294. doi: 10.1038/s41598-023-34602-0 (PMC10163253; doi:10.1038/s41598-023-34602-0)
Supplement: Supplementary file 1 — Supplementary Figures. [file 41598_2023_34602_MOESM1_ESM.docx]

**Supplementary Figure legends**

**Supplementary Fig. 1** **Differences in clinical features between the 3 clusters.**

**A-C.** Aneuploidy score, homologous recombination deficiency score, and intratumor heterogeneity between 3 clusters in the TCGA-LIHC cohort. **D.** Loss of heterozygosity score between 3 clusters in the TCGA-LIHC cohort. **E-G.** Ploidy score, TMB, and purity-related difference between 3 clusters in the TCGA-LIHC cohort. **H**. Differences in the proportion of 5 subtypes in 3 clusters. **I.** Genomic mutation landscapes between 3 clusters.

**Supplementary Fig. 2 Differences in clinical features among the 3 clusters**

**A.** The DEGs between C1 and C3 clusters were significantly enriched in 14 signaling pathways in the TCGA-LIHC cohort and 30 signaling pathways in the ICGC cohorts. **B.** The DEGs between C1 vs c2, C1 vs C3, and C2 vs C3 were significantly enriched in cell cycle and immune activation-related signaling pathways.

**Supplementary Fig. 1**


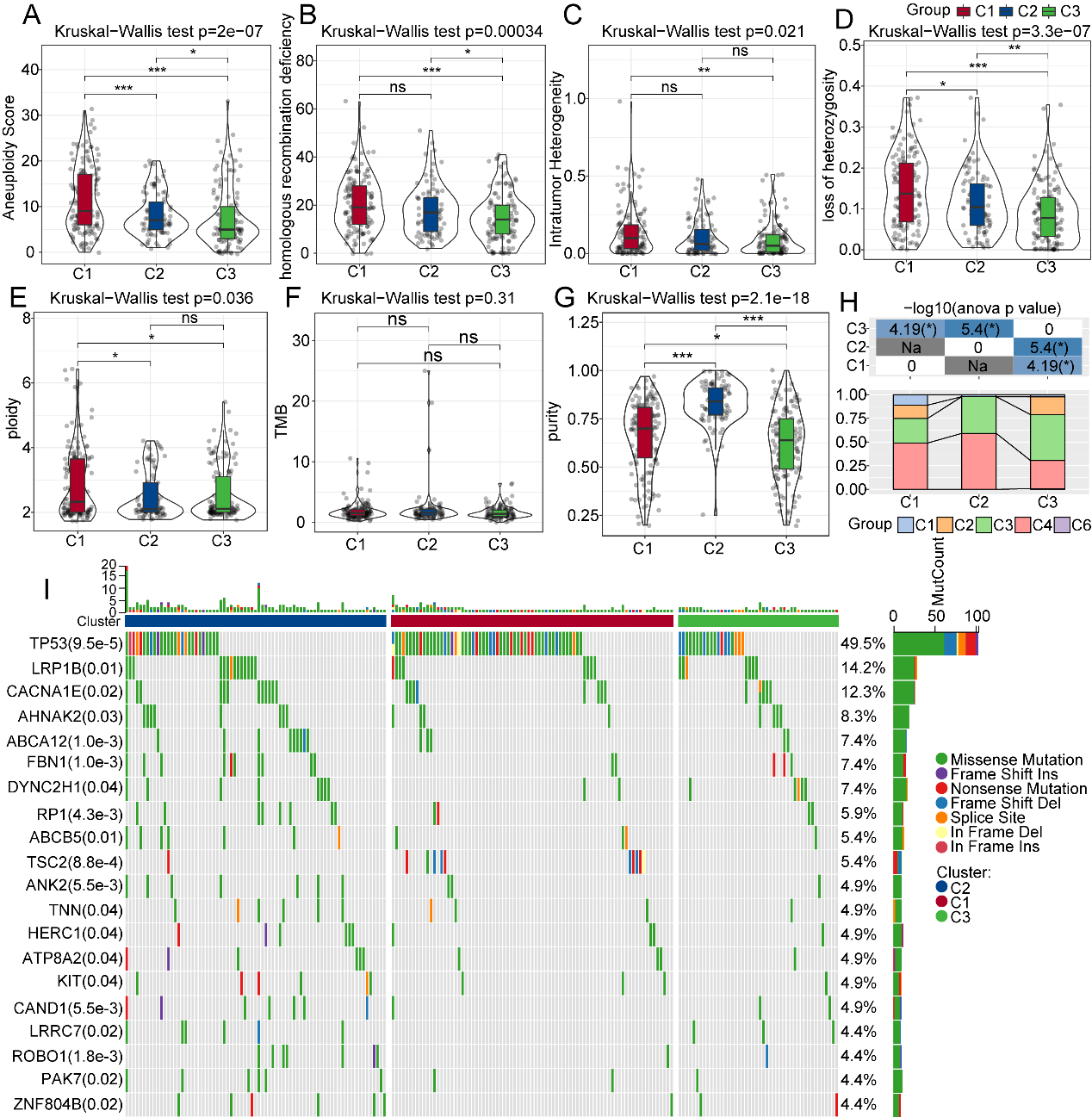


**Supplementary Fig. 2**

**
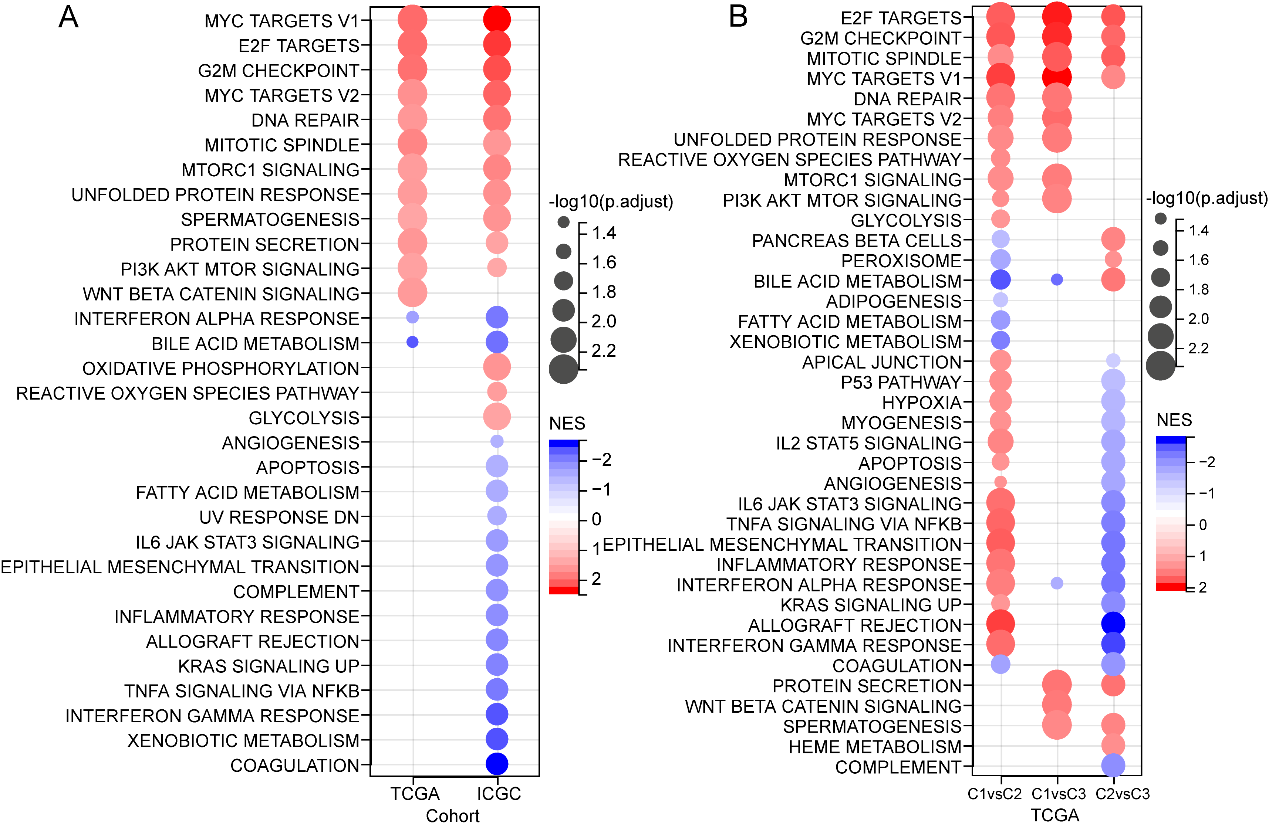
**
