# Supplementary material for: NK cell marker gene-based model shows good predictive ability in prognosis and response to immunotherapies in hepatocellular carcinoma
Source: Sci Rep. 2023 May 5;13:7294. doi: 10.1038/s41598-023-34602-0 (PMC10163253; doi:10.1038/s41598-023-34602-0)
Supplement: Supplementary file 2 — Supplementary Table S1. [file 41598_2023_34602_MOESM2_ESM.docx]

Supplementary Table 1. The information of primers sequences for qRT-PCR assay.

| Primer name | Sequence (5'-3') |
| --- | --- |
| GAPDH-R | GTCATGAGTCCTTCCACGATACC |
| GAPDH-F | GGAGTCCACTGGCGTCTTCA |
| CDC20-R | GGTGCCCACAGCCAAGTAGTTG |
| CDC20-F | CGTTACATTCCTTCCCTGCCAGAC |
| HMOX1-R | TGAGTGTAAGGACCCATCGGAGAAG |
| HMOX1-F | TGCCAGTGCCACCAAGTTCAAG |
| S100A9-R | GGTTAGCCTCGCCATCAGCATG |
| S100A9-F  CFHR3-R  CFHR3-F  PON1-R  PON1-F  GZMA-R  GZMA-F | AGAGACCATCATCAACACCTTCCAC  GGCATTGGTACTCGACTCTTGACTG  CTCTACAGAAGTTGCCTGCCATCC  AGACAACATACGACCACGCTAAACC  GGTGAACCATCCAGATGCCAAGTC  AAAGGGCTTCCAGAATCTCCATTGC  ACCAGGAACCATGTGCCAAGTTG |
